# Genome-wide association studies dissect the genetic architecture of seed and yield component traits in cowpea (*Vigna unguiculata* L. Walp)

**DOI:** 10.1093/g3journal/jkaf024

**Published:** 2025-02-07

**Authors:** Habib Akinmade, Rebecca Caroline Ulbricht Ferreira, Mario Henrique Murad Leite Andrade, Claudio Fernandes, Pablo Sipowicz, María Muñoz-Amatriaín, Esteban Rios

**Affiliations:** Plant Breeding Graduate Program, University of Florida, Gainesville, FL 32611, USA; Molecular Biology and Genetic Engineering Center, University of Campinas, Campinas, SP 13083-875, Brazil; School of Food and Agriculture, University of Maine, Orono, ME 04469, USA; Agronomy Department, University of Florida, Gainesville, FL 32611, USA; Plant Breeding Graduate Program, University of Florida, Gainesville, FL 32611, USA; Departamento de Biología Molecular (Área Genética), Universidad de León, León 24071, Spain; Agronomy Department, University of Florida, Gainesville, FL 32611, USA

**Keywords:** GWAS, image-based phenotyping, seed morphology, *Vigna unguiculata*, grain yield

## Abstract

The identification of loci related to seed and yield component traits in cowpea constitutes a key step for improvement through marker-assisted selection (MAS). Furthermore, seed morphology has an impact on industrial processing and influences consumer and farmer preferences. In this study, we performed genome-wide association studies (GWAS) on a mini-core collection of cowpea to dissect the genetic architecture and detect genomic regions associated with seed morphological traits and yield components. Phenotypic data were measured both manually and by high-throughput image-based approaches to test associations with 41,533 single nucleotide polymorphism markers using the FarmCPU model. From genome-associated regions, we also investigated putative candidate genes involved in the variation of the phenotypic traits. We detected 42 marker-trait associations for pod length and 100-seed weight, length, width, perimeter, and area of the seed. Candidate genes encoding leucine-rich repeat-containing (LRR) and F-box proteins, known to be associated with seed size, were identified; in addition, we identified candidate genes encoding PPR (pentatricopeptide repeat) proteins, recognized to have an important role in seed development in several crops. Our findings provide insights into natural variation in cowpea for yield-related traits and valuable information for MAS breeding strategies in this and other closely related crops.

**Core Ideas**

1. The traits exhibited high broad-sense heritability, particularly seed morphology traits, with estimates ranging from 0.93 to 0.97.
2. High correlation coefficient among seed morphology traits shows potential for indirect selection.
3. GWAS identified 42 genomic regions associated with seed and yield component traits.
4. Putative candidate genes responsible for the SNP-trait associations encode proteins involved in seed development and related processes.
5. A region on chromosome 3 is a hotspot associated with 6 traits that include important genetic regulators.

## Introduction

Cowpea (*Vigna unguiculata* L. Walp) is a highly versatile annual legume with grain, forage, and ornamental uses. It is an excellent source of protein (average of 25% protein in dried grain), and having a significant role in the diet in Sub-Saharan Africa and Brazil (Singh *et al.* 2006; Jayathilake *et al.* 2018; Mekonnen *et al.* 2022). In addition, significant variation in phenology (Dareus *et al.* 2021; Paudel *et al.* 2021; Andrade *et al.* 2023) and nitrogen fixation ability make cowpea a suitable option as a cover crop (Sánchez-Navarro *et al.* 2019; Nyaga and Njeru 2020). Cowpea is a diploid (2*n* = 2*x* = 22) species showing a broad gene pool (Lonardi *et al.* 2019; Muñoz-Amatriaín *et al.* 2021), and that is adapted to high temperatures and drought conditions, which are increasingly frequent due to global climate change (Agbicodo *et al.* 2009; Carvalho *et al.* 2017; Mohammed *et al.* 2024).

The main form of consumption of cowpea is as dried grain (Abebe and Alemayehu 2022), therefore, seed attributes such as size, color, and shape, are important traits for farmers, consumers, and the processing industry. Cowpea seed size is usually measured based on the weight of 100 seeds, and it shows a broad variation with values ranging from less than 10 g up to 30 g/100 seeds (Ehlers and Hall 1997). Cowpea seed size is classified into 3 groups: small (<12 g/100 seeds), medium (12–18 g/100 seeds), and large (>18 g/100 seeds) (Drabo *et al.* 1984). Eastern African countries prefer larger seeds (Mishili *et al.* 2009), and medium to large seeds are the most consumed in Brazil (Araújo 1988). Seed sizes significantly impact grain yield and it is a critical yield component (Manggoel *et al.* 2012; Aliyu and Makinde 2016). Furthermore, a positive correlation between seed length/area and cooking time has already been reported for cowpea (Traore *et al.* 2022). However, this relationship can also be influenced by the texture of the seed coat, as rough seeds tend to absorb water quicker, resulting to faster cooking times when compared to those with smoother seed coats (Muñoz-Amatriaín *et al.* 2021). Fast-cooking grains can also bring benefits to the environment and household habits (Diaz et al. 2021). Cowpea seed size preferences vary across different regions; factors such as cooking time, nutritional content, and local culinary culture greatly influence them. These preferences are key considerations for breeding programs as they determine the success of cowpea varieties for commercialization. For instance, in Nigeria, larger seed sizes are preferred for their appealing appearance. However, the extended cooking time required for these larger seeds can be a key disadvantage. Local practices such as the addition of “kanwa” (a type of rock salt) are usually employed to reduce cooking time (OECD 2019). Similarly, in Ghana, there is a general preference for larger-seeded cowpeas even though most of the locally grown cultivars have small seeds (Egbadzor *et al.* 2013).

In Burkina Faso, seed size preferences are closely linked to the seed's biochemical composition. Larger seeds are preferred for their higher starch concentrations, which is suitable for some cooking applications, while smaller seeds are valued for their higher phenolic content, associated with health benefits (Sombié *et al.* 2021). This dual preference explains diverse uses of cowpea in Burkinabe cuisine and the need for different breeding targets. Malawi presents another interesting case where larger seed sizes are preferred despite the longer cooking times required. This has led to a focus in breeding programs on developing large seed varieties with shorter cooking duration, balancing consumer preferences with cooking considerations (Chipeta *et al*. 2024). These regional preferences reiterate the importance of targeted breeding programs that consider local consumer habits, cooking practices, and nutritional needs. It also sheds light on the geographical areas where the different seed sizes are most prevalent and available.

Grain yield is a complex trait that is highly affected by genotype, environment, and their interaction (Li *et al.* 2020; Dareus *et al.* 2021; Rizzo *et al.* 2022). A fundamental requirement for the selection and cultivation of improved cowpea varieties is the estimation of the heritability and the correlation between yield and its related traits. Accurate estimation of heritability and trait correlations is important for effective selection and improvement of cowpea varieties. Owusu *et al*. (2021) reported that yield component traits such as the number of pods per plant, pod length, and the number of seeds per pod were positively correlated with grain yield. Previous studies showed that the estimated broad-sense heritability (*H*²) for different yield components in cowpea ranged from 0.11 to 0.96 and, in addition, significant positive correlations were reported between yield components (Shimelis and Shiringani 2010; Aliyu and Makinde 2016; Dareus *et al.* 2021; Pessoa *et al.* 2023). For instance, an increment in the number of pods per plant could increase total grain yield (Aliyu and Makinde 2016). In this scenario, identifying the loci and genes associated with grain yield components and seed traits will allow the development of genomic tools that can assist breeders in the selection of superior cultivars and predict genetic improvement for target traits (Tayade *et al.* 2023).

Advances in next-generation sequencing technologies have made it possible to generate genomic data at significantly faster rates and lower costs (Varshney *et al.* 2009). Regarding advances in genomic tools applied to cowpea, the development of the Cowpea iSelect Consortium Array (Illumina, Inc.), which comprises 51,128 single nucleotide polymorphisms (SNPs) (Muñoz-Amatriaín *et al.* 2017), increased the capacity for genetic research in the species (Xu *et al.* 2017). This set of SNPs has been used in genomic studies in cowpea, including genome-wide association studies (GWAS) in a mini-core collection developed at the University of California, Riverside: the UCR Minicore (Muñoz-Amatriaín *et al.* 2021; Paudel *et al.* 2021; Andrade *et al.* 2023). This valuable collection contains 368 domesticated accessions of cowpea that represent the genetic variability of approximately 5,000 cowpea accessions (Muñoz-Amatriaín *et al.* 2021). Moreover, the use of high-throughput phenotyping (HTP) encompassing image acquisition has significantly increased the effectiveness of genetic studies in important crops. The use of HTP enables the screening of large mapping populations, germplasm collections, and other breeding resources. This approach increases precision and accuracy in measuring several phenotypic traits, including seed morphology (Mir *et al.* 2019; Giordani *et al.* 2022).

Association studies in cowpea have been reported for traits related to phenology such as time to flowering (Seo *et al.* 2020; Muñoz-Amatriaín *et al.* 2021; Paudel *et al.* 2021; Andrade *et al.* 2023), pod maturity (Andrade *et al.* 2023), root architecture (Burridge *et al.* 2017), pod length (Xu *et al.* 2017), pod load score, and dry pod weight (Muñoz-Amatriaín *et al.* 2021). Association studies also cover seed weight, width, length and density (Lo *et al.* 2019), protein content (Chen *et al.* 2023), and resistance to biotic (Bhattarai *et al.* 2017; Miesho *et al.* 2019; Dong *et al.* 2022; Kpoviessi *et al.* 2022) and abiotic stresses (Wu *et al.* 2021; Ravelombola *et al.* 2022). Regarding cowpea seed size, Lo *et al*. (2019) assessed the associations between 51,128 SNP markers and 4 seed traits and identified 17 significant loci. However, there are still no studies on combining GWAS with high-density SNPs and HTP to dissect cowpea seed morphology. There is a growing interest in understanding the genetic architecture of seed morphology and yield component traits, aiming to select plant varieties that combine traits such as large seed, adaptation to industrial processing, consumer appeal, and high yield (Blair *et al.* 2009; Lei *et al.* 2020), which is vital for food security and sustainable agriculture (Aliyu and Makinde 2016; Rizzo *et al.* 2022).

The goal of this study was to perform GWAS on the UCR Minicore (Muñoz-Amatriaín *et al.* 2021) to study the genetic architecture and identify genomic regions associated with grain yield components and seed morphology traits. In addition, we explored the identified genomic regions in search of candidate genes putatively associated with the phenotypic traits evaluated.

## Materials and methods

### Plant materials and field design

Accessions from the UCR Minicore collection (Muñoz-Amatriaín *et al.* 2021) and 10 checks were used in this study, totaling 375 accessions. The field experiment was carried out in 2021 at the Plant Science Research and Experimental Unit (PSREU), Citra, FL (29.4119°N; 82.1098°W), using a row-column design with 2 replicates. Each experimental unit (3 m × 0.6 m) consisted of 10 plants manually seeded and spaced at 0.3 m within row and 0.6 m between row spacing.

### Phenotypic evaluation

To evaluate all the traits, 3 plants were harvested from the center of each plot to prevent the border effect. At harvest, the evaluation of pod characteristics was conducted to determine the number of pods per plant and the average length of the pods. Detailed measurement methods for each of the traits are explained in Table 1.

**Table 1.** List of cowpea seed and pod traits evaluated in the present study.

Character	Traits	Unit	Method	Description
Pod	Pod length	cm	1	Longitudinal measurement of 5 randomly chosen mature pods
	Pod number	pods	1	Average number of pods counted on 3 plants at harvest
Seeds	100-seed weight	g	1	Average 100-seed weight from 3 plants per plot
	Grain yield	g	1	Mean seed yield collected from the 3 plants harvested at pod maturity stage per plot
	Seed length	mm	2	SmartGrain software
	Seed width	mm	2	SmartGrain software
	Seed perimeter	mm	2	SmartGrain software
	Seed area	mm²	2	SmartGrain software

Each trait's measurement method is indicated by a code: 1 for manual measurements and 2 for measurements taken from digital images.

**Fig. 1.**
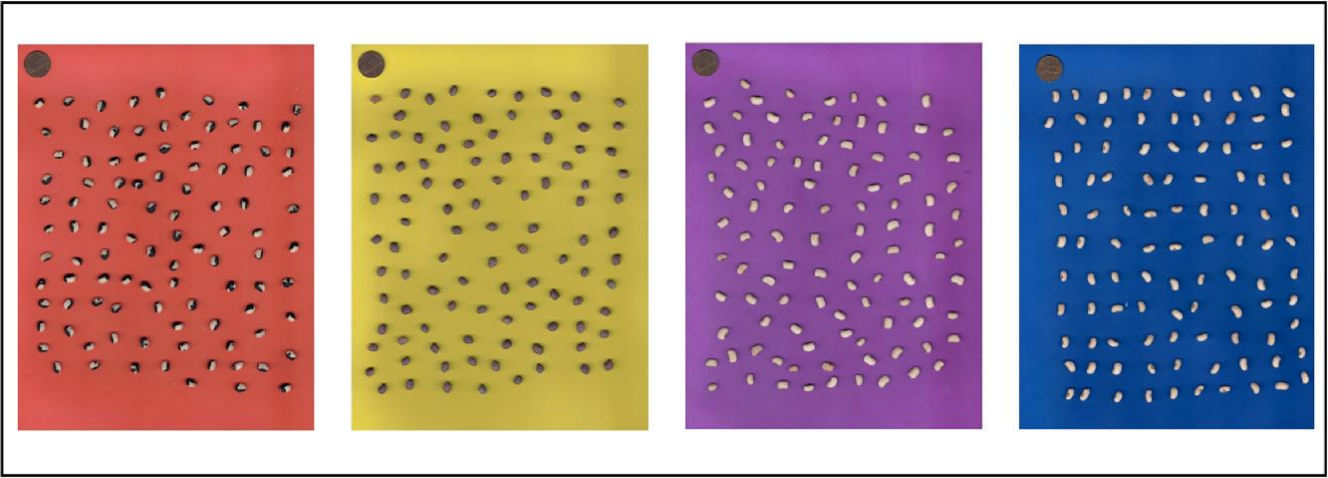
Cowpea seeds placed on different backgrounds used for high-throughput image-based phenotyping for seed morphology traits (one cent coin placed above for size reference).

Traits related to seed size and shape (area, perimeter, length, and width) were assessed through a high-throughput process using seed images. One hundred seeds per plot were scanned using a scanner (Xerox VersaLink C405). Four background colors (red, yellow, purple, and blue) were used to better contrast the seed color (Fig. 1). The SmartGrain software (Tanabata *et al.* 2012) was used to process and analyze the images. After processing the images, SmartGrain detects every seed and measures its shape using different parameters (Giordani *et al.* 2022).

### Statistical analysis for the phenotypic data

The adjusted means (best linear unbiased estimates—BLUEs) of the accessions for each of the traits were estimated using the following linear mixed model:


(1) $$y=1\mu +X\beta +Z_{1}r+Z_{2}c+\varepsilon$$

where y is the vector of the phenotypic data, *μ* is the overall mean, β is the vector of the fixed effects of the accessions, r and c are the random effects of the rows and columns with $r∼N(0,\sigma_{r}^{2})$ and $c\;∼N(0,\sigma_{c}^{2})$; $\sigma_{r}^{2}$ and $\sigma_{c}^{2}$ are the variance components associated with the effect of rows and columns; ε is the vector of the residuals, with $\varepsilon ∼N(0,\sigma_{e}^{2})$; and $\sigma_{e}^{2}$ is the variance component of the residuals. *X* is the design matrix of the fixed effects; and $Z_{1}\;$ and $Z_{2}\;$ are the design matrix of the effects of rows and columns, respectively.

A second model considering accessions as random effects was used to estimate the broad-sense heritability for all the traits following the formula: $H^{2}\;=\frac{\sigma_{g}^{2}}{\left(\sigma_{g}^{2}\;+\;\frac{\sigma_{e}^{2}}{r}\right)}$, where $\sigma_{g}^{2}$ is the genotypic variance, $\sigma_{e}^{2}$ is the error variance, and *r* is the number of replications.

Genetic correlation between pairs of traits was calculated using the following bivariate model that accounts for both fixed and random effects in the data:


(2) $$y\;=\;X\beta +Z_{1}r+Z_{2}c+Z_{3}a+\varepsilon$$

where y is a stacked vector of the phenotypic data for pair of traits (*t*1 and *t*2), β is the stacked vector of the fixed effects of the intercept for each trait, *r* is the random effect stacked vector of the row for each trait, $r∼N(0,I_{h}⊗I\sigma_{r}^{2})$, *c* is the random effect stacked vector of the column for each trait, $c∼N(0,I_{h}⊗I\sigma_{c}^{2})$, *a* is the random effect stacked vector of the accessions for each trait, $a∼N(0,I_{a}⊗G)$, $G=[\sigma_{at1}^{2}\;\sigma_{at1t2}^{2}\;\sigma_{at1t2}^{2}\;\sigma_{at2}^{2}\;]$, and ε is the random effect stacked vector of the accessions for each trait, $\varepsilon ∼N(0,I_{e}⊗R)$, $R=[\sigma_{et1}^{2}\;\sigma_{et1t2}^{2}\;\sigma_{et1t2}^{2}\;\sigma_{et2}^{2}\;]$. *X* is the design matrix of the fixed effects; Z1, Z2, and Z3 are the design matrix of the effects of rows, columns, and accessions, respectively. The components $\sigma_{r}^{2}$, $\sigma_{c}^{2}$, $\sigma_{at1}^{2}$, $\sigma_{at2}^{2}$, $\sigma_{et1}^{2}$, and $\sigma_{et2}^{2}$ are the variance components for row, column, accessions for trait 1, accessions for trait 2, error for trait 1, and error for trait 2. We also calculated the pairwise phenotypic correlation between all traits. All analyses were conducted using the ASReml-R package (Butler *et al.* 2023) within the R statistical software (R Core Team 2023). Broad-sense heritability and genetic correlations were calculated using the variance values from the output of same package. Pairwise phenotypic correlations between traits were calculated in R using the cor() function with the Pearson method.

### Genotypic data

Genotyping was carried out as described by Muñoz-Amatriaín *et al*. (2017, 2021), Paudel *et al*. (2021), and Andrade *et al*. (2023). Briefly, the 375 cowpea accessions were genotyped at the University of Southern California Molecular Genomics Core (Los Angeles) using the Cowpea iSelect Consortium Array, with 51,128 SNPs. SNPs were called with GenomeStudio (Illumina Inc.) and their physical positions were determined using the IT97K-499-35 reference genome v1.0 (Lonardi *et al.* 2019). Loci were filtered to remove high levels (>25%) of missing data and to avoid false positives by using a minor allele frequency threshold of 5%. This resulted in a final set of 41,533 SNPs for the subsequent analyses.

### Genome-wide association study

We used GWAS to identify genomic regions controlling yield components and seed traits in the UCR Minicore. SNP-trait association analyses were performed using the GAPIT package (Lipka *et al.* 2012) in R (R Core Team 2023) using the FarmCPU multivariate method (fixed and random model circulating probability unification) due to its strong ability to control false positives while using both fixed and random effect models in iteration (Liu *et al.* 2016). We also used an additional MLM approach which incorporates population structure and kinship in controlling confounding factors (Yu et al. 2006). The results from both methods were compared to check the consistency of SNP-trait associations. The first 6 principal components (PCs) were calculated and employed as a covariate to account for the population structure (Andrade *et al.* 2023). Subsequently, Manhattan and QQ plots were generated using *ggplot2* and *qqman* R package (Wickham 2016; Turner 2018; R Core Team 2023). The phenotypic variance explained (PVE) was calculated following the equation described by Giordani *et al*. (2022): *V*_qtl_ = 2freq × (1–freq) × effect2, where *V*_qtl_ equals the variance of the QTL effect and freq is the allele frequency. This calculation was based on the variance of the adjusted means for each trait (*V*_pheno_).

### Identification of candidate genes

Based on the linkage disequilibrium (LD) decay for the sample set analyzed in this study and following the method used by Andrade *et al*. (2023), we started from the physical position of the significant SNP markers and we extracted annotated gene models within a window of 10 kb upstream to 10 kb downstream in the cowpea reference genome IT97K-499-35 v1.2 (Liang *et al.* 2024). From the gene annotations, gene ontology (GO) terms (Ashburner *et al*. 2000)—molecular functions, cellular component, and biological process—were retrieved and summarized using the REViGO tool (Supek *et al.* 2011). Furthermore, we consulted the Legume Information System website (LIS, https://mines.legumeinfo.org/vignamine/begin.do) to assess the expression patterns of the annotated genes during seed and pod development and we select only genes potentially related to the phenotypes analyzed in our study.

## Results

### Phenotypic analysis

**Fig. 2.**
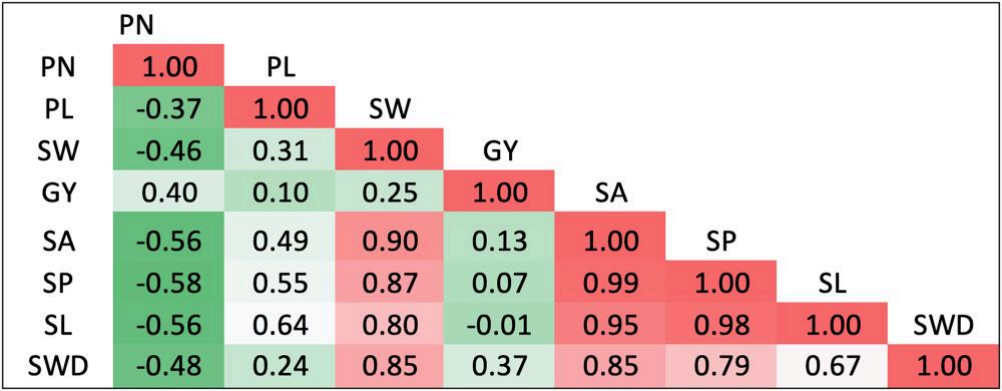
Genetic correlation among 8 seed and yield component traits of the UCR Minicore collection in this study. PN, pod number; PL, pod length; SW, 100-seed weight; GY, grain yield; SA, seed area; SP, seed perimeter; SL, seed length; SWD, seed width.

The UCR Minicore cowpea collection revealed a wide phenotypic variation across the traits evaluated in this study (Table 2 and Fig. 2). The CV for all traits was medium to high, ranging from 12.3 (seed width) to 29.6 (grain yield). Broad-sense heritability estimates (*H*^2^) were high for all the traits, and moderate for grain yield and pod number. Six traits (seed area, pod length, seed length, seed perimeter, 100-seed weight, and seed width) showed heritability estimates greater than 0.9, ranging from 0.93 (pod length and seed width) to 0.97 (seed length), while broad-sense heritability for grain yield and the number of pods was 0.51 (Table 2).

**Table 2.** Summary of mean (with range), standard deviation (SD), broad-sense heritability (*H*²), and coefficient of variation (CV) for 8 traits evaluated in the cowpea mini-core collection in Citra, FL.

Trait	Mean	SD	*H* ^2^	CV
Area (mm^2^)	36.7 (8.1–75.2)	9.3	0.95	24.6
Pod length (cm)	17.2 (9.8–42.5)	4.5	0.93	24.8
Seed length (mm)	8.3 (4.1–11.9)	1.2	0.97	14.6
Number of pods/plant	25.9 (4.0–68.3)	10.5	0.51	26.6
Seed perimeter (mm)	23.6 (11.0–33.9)	3.3	0.96	13.7
100-seed weight/plant (g)	15.9 (6.7–30.1)	4.1	0.94	24.9
Seed width (mm)	5.6 (2.8–8.1)	0.7	0.93	12.3
Grain yield (g)	31.4 (4.2–85.9)	13.7	0.51	29.6

Traits followed a normal distribution, as determined through both visual inspection (Supplementary Fig. 1) and the Shapiro–Wilk test, which is desired for the dissection of the genetic architecture of quantitative traits (Zeng *et al.* 2022). We also evaluated genetic and phenotypic correlation coefficients among traits (Fig. 2 and Supplementary Fig. 1), and both correlations have consistent results for all trait pairs. The number of pods had negative correlations with pod length, seed area, seed length, and seed perimeter, and a positive correlation with grain yield. Correlation coefficients among the rest of the traits were positive, with emphasis on positive correlations between seed size traits (Fig. 2). The highest correlation coefficients were found between seed perimeter and seed area, seed perimeter and seed length, and seed area and seed length for genotypic (0.99, 0.98, and 0.95, respectively) and phenotypic (0.99, 0.98, and 0.94, respectively) estimates.

### GWAS analysis

**Fig. 3.**
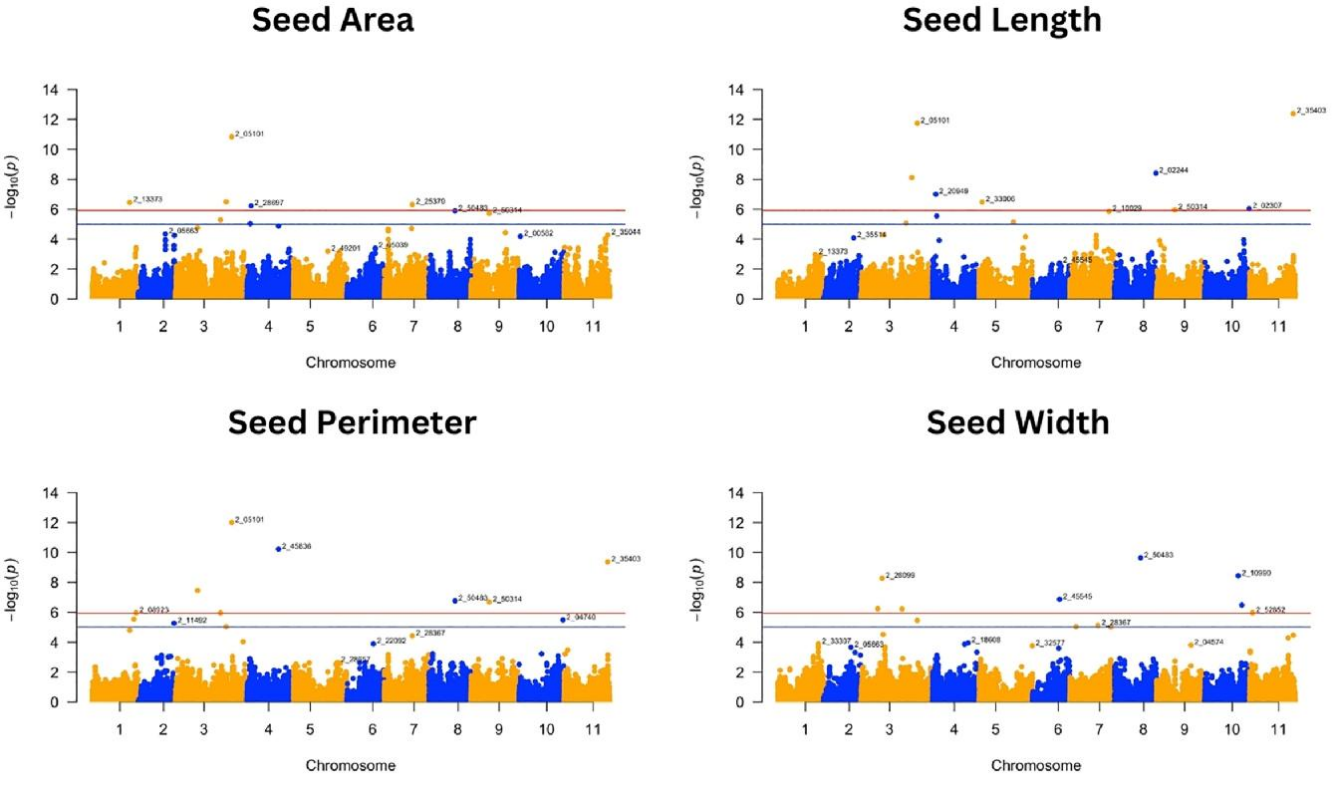
Manhattan plots of GWAS for seed traits: seed area, seed length, seed perimeter, and seed width. Negative log_10_(*p*) values are plotted against physical position on each of the 11 chromosomes (Lonardi *et al*. 2019). The dashed line at the higher significance threshold indicates the Bonferroni correction for genome-wide significance threshold (5.92).

**Fig. 4.**
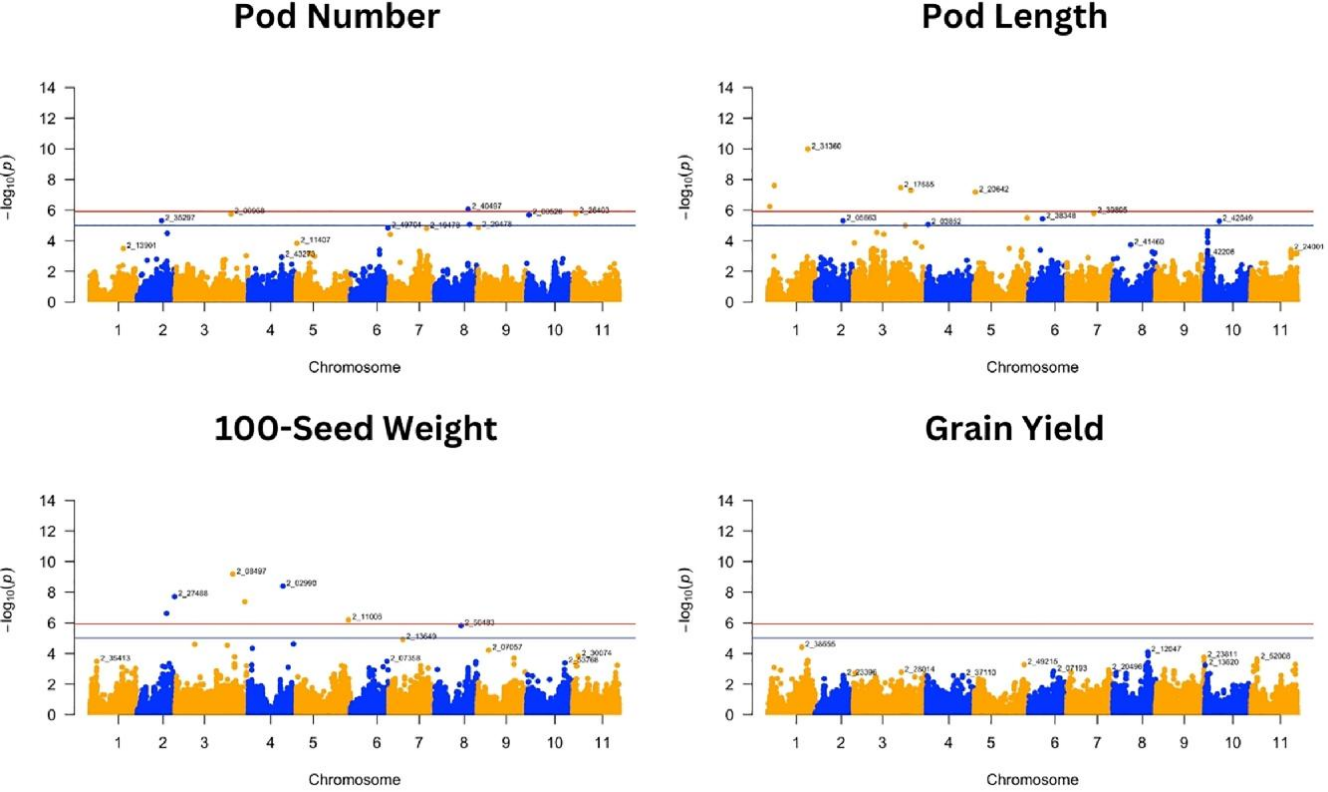
Manhattan plots of GWAS for pod number, pod length, 100-seed weight, and grain yield. Negative log_10_(*p*) values are plotted against physical position on each of the 11 chromosomes. The dashed line at the higher significance threshold indicates the Bonferroni correction for genome-wide significance threshold (5.92).

For the 8 traits analyzed, GWAS resulted in 37 SNPs that were significantly associated with 7 traits following the Bonferroni correction threshold (∼5.92) (Figs. 3 and 4 and Table 2). GWAS did not find any significant SNP associated with grain yield. Significant SNPs were distributed across the 11 cowpea chromosomes, with a higher abundance of SNPs detected on chromosome 3 (Table 3). The SNP alleles in Table 3 are referenced relative to the Watson strand (Liang *et al*. 2024), as determined using the Cowpea iSelect array SNP dataset (Supplementary Tables 2 and 3). These tables provide SNP positions across different genome assemblies and their corresponding reference and alternate alleles.

**Table 3.** GWAS summary of trait, SNP identification, chromosomal locations (Chr), *P*-values, minor allele frequency (MAF), effects, effect allele (based on Watson strand; Liang *et al.* 2024), and percentage of phenotypic variance explained (PVE %).

Traits	SNP	Chr	Position	*P*-value	MAF	Effect	Effect allele	PVE (%)
**Pod number**	2_40497	8	30,008,951	8.70E-07	0.46622	2.4615	C	2.85
**100-seed weight**	2_00066	2	26,110,941	2.39E-07	0.10270	−1.3039	T	0.30
	2_27488	2	33,282,856	1.90E-08	0.22432	−1.0489	T	0.36
	2_08497	3	51,450,779	6.62E-10	0.24054	−1.1995	G	0.50
	2_16470	3	62,141,166	4.20E-08	0.43514	0.8946	G	0.37
	2_02990	4	30,912,835	4.06E-09	0.13514	−1.2131	G	0.32
	2_11006	5	46,857,366	6.32E-07	0.39865	−0.6961	G	1.37
**Pod length**	2_00801	1	1,330,630	5.70E-07	0.07162	1.7181	C	2.20
	2_31360	1	35,353,094	1.05E-10	0.06216	2.0758	A	2.81
	2_38893	1	5,453,084	2.48E-08	0.14054	−0.8709	A	1.05
	2_17685	3	42,267,565	3.46E-08	0.34324	−0.7879	A	1.68
	**2_05101**	**3**	**51,419,219**	**5.16E-08**	**0**.**24189**	**0**.**8241**	**A**	**1**.**49**
	2_20642	5	1,274,892	6.73E-08	0.12703	−0.8374	C	0.82
**Seed area**	2_13373	1	34,263,930	3.53E-07	0.21757	−1.7665	C	5.36
	**2_10581**	**3**	**46,387,165**	**3.14E-07**	**0**.**27973**	**1**.**9274**	**G**	**7**.**49**
	**2_05101**	**3**	**51,419,219**	**1.44E-11**	**0**.**24189**	**2**.**9050**	**A**	**15**.**65**
	2_28697	4	3,852,847	5.88E-07	0.30000	−1.8118	T	6.92
	2_25379	7	25,611,501	4.88E-07	0.46892	2.4222	G	3.40
**Seed length**	2_02307	10	40,838,835	9.19E-07	0.36081	−0.2100	G	0.02
	**2_35403**	**11**	**39,825,657**	**4.06E-13**	**0**.**12432**	**−0**.**4402**	**A**	**0**.**05**
	**2_10581**	**3**	**46,387,165**	**7.54E-09**	**0**.**27973**	**0**.**2803**	**G**	**0**.**04**
	**2_05101**	**3**	**51,419,219**	**1.83E-12**	**0**.**24189**	**0**.**3692**	**A**	**0**.**06**
	2_20949	4	3,177,960	9.83E-08	0.22432	−0.2699	G	0.03
	2_33006	5	3,004,079	3.29E-07	0.20135	−0.2190	C	0.02
	2_02244	8	37,626,075	3.84E-09	0.45405	−0.3340	C	0.06
	**2_50314**	**9**	**16,653,792**	**1.07E-06**	**0**.**12297**	**−0**.**2854**	**T**	**1**.**19**
**Seed perimeter**	2_08923	1	39,971,764	1.08E-06	0.45676	−0.5763	T	10.90
	**2_35403**	**11**	**39,825,657**	**4.13E-10**	**0**.**12432**	**−0**.**9818**	**A**	**13**.**98**
	2_10911	3	20,318,916	3.56E-08	0.30405	−0.6636	T	12.44
	2_02917	3	41,213,480	1.11E-06	0.11486	−0.7260	G	7.03
	**2_05101**	**3**	**51,419,219**	**9.92E-13**	**0**.**24189**	**0**.**9820**	**A**	**23**.**74**
	2_45836	4	28,968,935	6.06E-11	0.24730	−0.9229	G	21.18
	**2_50483**	**8**	**23,709,814**	**1.72E-07**	**0**.**24189**	**0**.**9340**	**A**	**21**.**17**
	**2_50314**	**9**	**16,653,792**	**2.04E-07**	**0**.**12297**	**−0**.**8208**	**T**	**1**.**36**
**Seed width**	2_10990	10	30,818,600	3.67E-09	0.17568	−0.1794	T	0.09
	2_24784	10	34,158,765	3.31E-07	0.20541	0.1336	A	0.05
	2_52852	11	2,602,978	1.06E-06	0.09730	0.1621	G	0.04
	2_51099	3	15,403,007	5.85E-07	0.11892	−0.1742	C	0.06
	2_26099	3	19,380,766	5.29E-09	0.14595	0.2844	A	0.18
	2_21078	3	37,535,846	6.00E-07	0.37162	0.1205	C	0.06
	2_45545	6	24,933,702	1.38E-07	0.09595	−0.1724	C	0.05
	**2_50483**	**8**	**23,709,814**	**2.32E-10**	**0**.**24189**	**0**.**2363**	**A**	**3**.**95**

SNPs associated with multiple traits are bolded.

Pod number was associated with only 1 SNP, while seed length, seed perimeter, and seed width were those associated with 8 SNPs. Out of the 42 associations, 5 SNPs, bolded in Table 3, were associated with multiple traits. We detected 1 SNP in common between pod length, seed area, seed length, and seed perimeter (2_05101), 1 SNP in common for seed area and seed length (2_10581), 1 SNP in common for seed perimeter and seed width (2_50483), and 2 SNPs in common for seed perimeter and seed length (2_35403; 2_50314) (Table 3). It is worth noting that the “2_” prefix in the marker names does not indicate chromosome location, as these markers are distributed across multiple chromosomes in the genome.

Seed weight was associated with 6 SNPs located on chromosomes 2, 3, 4, and 5, which individually were responsible for the PVE from 0.30 to 1.37%. Pod length had 6 SNPs distributed on chromosomes 1, 3, and 5, which explained a total phenotypic variation of 10.03%. The seed area had SNPs distributed on chromosomes 1, 3, 4, and 7, explaining a total PVE of 38.82%, while seed length had 8 significant SNPs distributed on chromosomes 3, 4, 5, 8, 10, and 11 that explained a total PVE of 1.47%. Eight SNPs on chromosomes 1, 3, 4, 8, 9, and 11 explained from 1.36 to 23.74 of PVE for seed perimeter trait, and seed width had significant SNPs distributed on chromosomes 3, 6, 8, 10, and 11 that explained a total PVE of 4.48% (Table 3).

Manhattan (Figs. 3 and 4) and QQ plots (Supplementary Fig. 2) are presented for all traits to aid with the verification of the SNP markers that are significantly associated with the different traits. The QQ plots revealed that the observed and expected values were largely matched, suggesting that the GWAS results controlled for the effects of population structure. While FarmCPU was able to detect several marker-trait associations, the MLM approach detected a single SNP (Supplementary Figs. 4 and 5), which was for pod length (2_31360) on chromosome 1, equally detected using the FarmCPU approach.

### Candidate genes

Genes within 10 kb regions flanking significant SNPs were identified using the IT97K-499-35 v1.2 reference genome (Liang *et al*. 2024). A total of 63 candidate genes were found within the regions examined, 37 of which could be assigned to GO terms. According to LIS dataset, all 63 candidate genes were expressed in 1 or more tissues or stages and 12 were considered the most promising based on expression patterns. All genes identified, as well as their functional annotations and associated GO terms, are included in Supplementary Table 1.

For 100-seed weight, 10 candidate genes were detected, among which there was a F-box/RNI-like superfamily protein (*Vigun02g197700—*SNP 2_27488) and a leucine-rich repeat (LRR)-containing protein (*Vigun03g320300—*SNP 2_08497). We identified 2 genes for pod number (*Vigun08g129300* and *Vigun08g129400—*SNP 2_40497) encoding exocyst subunit exo70 family protein B1, in addition to 13 candidate genes for pod length, among which there was a protein-tyrosine-phosphatase (*Vigun05g016000*—SNP 2_20642) and a tetratricopeptide repeat (TPR)-like superfamily protein (*Vigun03g256400—*SNP 2_17685). Eighteen candidate genes were identified for seed length, including *Vigun10g194600* (zinc finger protein 3—SNP 2_02307) and *Vigun04g037900* (pentatricopeptide repeat (PPR) superfamily protein—SNP 2_20949). For seed width, we detected 13 candidate genes, among which there was a lipid transfer protein (*Vigun03g167100—*SNP 2_26099) and a gibberellin 20 oxidase 2-like (*Vigun03g149400—*SNP 2_51099) (Supplementary Table 1).

As shown in GO categories in Supplementary Fig. 3, the candidate genes were assigned to GO terms in 3 classes: molecular function, cellular component, and biological processes. Catalytic activity (GO:0003824), binding (GO:0008152), and metabolic processes (GO:0008152) were the most represented subcategories. Some candidate genes were associated with more than 1 function (Supplementary Table 1). For instance, *Vigun05g279200* (SNP 2_11006) was annotated as associated with hydrolase activity (GO:0016787) and lipid metabolic process (GO:0006629), while *Vigun10g108700* (SNP 2_10990) was associated with enzyme inhibitor activity (GO:0004857) and pectinesterase activity (GO:0030599) (Supplementary Table 1).

## Discussion

### Genetic basis of seed and yield components in cowpea

Cowpea is a multifunctional legume with the potential to contribute significantly to global food and nutritional security. Furthermore, it can be part of a sustainable food system, as it is a valuable genetic resource for agricultural sustainability under climate change conditions (Mekonnen *et al.* 2022). In this study, we considered several yield component traits (pod length, pod number, and 100-seed weight) and seed morphology traits (seed area, seed length, seed perimeter, and seed width) that are highly relevant in cowpea breeding programs. Some of those traits also have an impact on industrial processing and influence consumer and farmer preferences. Therefore, understanding the genetic mechanisms underlying these traits can provide valuable insights for breeders in developing superior cultivars with high yield and consumer acceptance (Giordani *et al.* 2022).

To assess the genetic architecture of those traits, we used most accessions in the UCR Minicore (Muñoz-Amatriaín *et al.* 2021) and 10 checks, totaling 375 accessions. In this study, seed size and shape traits were assessed using SmartGrain software, which processed images to detect individual seeds and measure their morphological parameters. The use of different background colors allowed for optimal contrast and accurate seed measurements. While we focused on average trait values derived from 100 seeds per replicate plot, variance heterogeneity mapping would be a complementary perspective for future studies, taking advantage of the software's ability to detect individual seeds. The approach, as demonstrated in studies like Hussain et al. (2020), can reveal genetic loci associated with phenotypic variability and plasticity. Previous studies using this collection observed wide genotypic and phenotypic diversity in pod and seed types and colors, flowering time, pod maturity, and plant architecture, among others (Dareus *et al.* 2021; Muñoz-Amatriaín *et al.* 2021; Andrade *et al*. 2023). Herein, significant genotypic variation was found for all traits, and high broad-sense heritability (>90%) was observed for almost all the traits measured, except the number of pods and grain yield. This aligns with previous studies, such as Mofokeng *et al.* (2020), who reported broad-sense heritability exceeding 90% for pod number, 100-seed weight, and seed per pod. Similarly, Owusu *et al*. (2021) and Kgasudi *et al*. (2024) observed heritability with >90% for 100-seed weight. The high heritability can also be linked to the precision of the HTP system (SmartGrain software) used, which minimizes human error and measurement variability in data collection. This method is particularly important for dimensional traits (for example: area, length, width, and perimeter of pods and seeds) as they are measured in good detail and highly accurate measurement (Tanabata *et al.* 2012). This method was also employed by Giordani *et al.* (2022) for evaluating seed dimensions of 180 common bean accessions. These findings are consistent with Araus *et al*. (2018), who emphasized how high-throughput methods improve the accuracy of genetic evaluations in plant breeding. Moreover, the high heritability estimates suggest that a significant portion of the observed phenotypic variation can be attributed to the genetic component, evidencing its relevance in the expression of these traits, and thus once again endorsing the usefulness of this Minicore for GWAS.

However, since the heritability values were estimated from variance components based on data from a single season, they may not fully capture the genotype-by-environment interactions (G×E). Multi-environment trials, such as comparing the performance of the UCR Minicore accessions across southern California (where it was developed) and Florida, could help evaluate G×E effects and validate the genetic variation observed. Despite this limitation, the high heritability estimates observed here, combined with the use of HTP methods, decreases the likelihood for errors, improving the reliability of the genetic differences observed. While the heritability estimates in this study are higher, they are also consistent with previous reports of high heritability for seed morphology traits in cowpea (Lo *et al*. 2019) and other crops, including common beans (*Phaseolus vulgaris* L.) (Wu *et al.* 2020; Giordani *et al*. 2022) and wheat (*Triticum aestivum* L.) (Schierenbeck *et al.* 2021). Future research should incorporate larger collections and multi-environment trials to validate these results and expand their application to breeding programs.

We observed high positive correlations between seed dimension traits (Fig. 2). Seed width was positively correlated with 100-seed weight and seed length, agreeing with the results of path coefficient analyses of cowpea by Walle *et al*. (2018). This implies that the increased seed width of cowpea plants tends to have a higher seed length and 100-seed weight. These correlations are important in breeding programs that seek to improve seed quality, as selections for 1 trait will lead to an improvement in the other seed dimensions, creating a more efficient method to improve the overall seed quality and yield potential. Expectedly, we detected a positive association for grain yield and the number of pods, corroborating with Chaudhary *et al*. (2020), which suggests that the increase in the pod number will contribute to higher yield. This is a key for plant breeding, as selecting cowpea for higher number of pods will directly increase the total yield that the plant will achieve. We also report a positive correlation between 100-seed weight and pod length, similar to Walle *et al*. (2018), suggesting that breeding for longer pods can indirectly lead to larger seeds, improving the seed weight. Conversely, the number of pods was negatively correlated with 100-seed weight and pod length, in agreement with reports by Meena *et al*. (2015). This negative association suggests a tradeoff, with an increase in seed size leading to lower number of pods the plant will produce, a strong consideration for breeders that might want to balance the seed size with the pod number for the best yields. From a breeding perspective, these significant associations are beneficial for the simultaneous improvement of multiple yield traits in cowpea. The positive associations observed between pod number, seed size, and yield show the potential for selecting multiple traits in a single breeding cycle. However, it is advisable to adopt breeding methods like marker-assisted selection (MAS) that can break undesirable linkages for the negatively correlated traits (Hasan et al. 2015). The knowledge of these associations will assist breeders in making better decisions in choosing parental lines that show favorable trait combinations while reducing the impact of negative correlations (Khan *et al.* 2022).

### SNP-trait association

Due to the availability of low-cost, high-throughput SNP genotyping methods and new genomic resources, GWAS has been used as an effective method to dissect the genetic architecture of target traits in plant breeding. Previous studies explored the wide genetic diversity of cowpea accessions from the UCR Minicore collection and identified genomic regions related to phenological characteristics (Muñoz-Amatriaín *et al.* 2021; Paudel *et al.* 2021; Andrade *et al.* 2023), seed coat color (Herniter *et al.* 2018; Herniter *et al.* 2024), seed size (Lo *et al.* 2019), and bruchid resistance (Miesho *et al.* 2019), demonstrating its appropriateness for GWAS. However, efforts are still needed to dissect the genetic mechanisms of seed and yield component traits in cowpea to devise marker-based breeding approaches and achieve greater genetic gains.

In the present study, we identified 37 genomic regions with small to moderate effects significantly associated with yield component traits (pod length, pod number, 100-seed weight, and yield) and seed morphology traits (seed area, seed length, seed perimeter, and seed width) in cowpea (Table 3). Previous studies with cowpea detected QTLs for some of the traits analyzed here, including pod length and seed weight (Pan *et al.* 2017; Lo *et al.* 2019). Notably, SNP 2_50483, located on chromosome 8 (23,709,814 bp), was previously associated with seed weight (Lo *et al.* 2019), suggesting its potential for MAS. Our study further expands on this by identifying additional regions associated with relevant characteristics in cowpea.

Three SNPs, 2_05101, 2_45836, and 2_50483, were significantly associated with seed perimeter and explained over 21% of the phenotypic variance. Interestingly, seed perimeter showed higher PVEs than other traits, which may be a pointer that loci controlling seed perimeter have relatively larger effects or less complex genetic interactions compared with other traits analyzed. These SNPs are promising candidates for further validation and utilization in MAS, especially since they were also associated with other traits (e.g. SNP 2_05101 for pod length, seed area, and seed length) evaluated in the present study (Table 3). Furthermore, it appears that chromosome 3 plays an important role in seed and pod phenotypes, corroborating with other studies (Lo *et al.* 2019; Wu *et al.* 2024). In MAS, pleiotropic effects should be considered, as improving 1 trait may have an influence on other correlated traits. For example, SNP 2_05101, associated with seed perimeter, also affects pod length, seed area, and seed length. These inter-trait relationships show the need for careful selection strategies to reduce undesirable trade-offs in a bid to improve target traits.

The MLM approach was able to identify much less significant SNPs due to its higher stringency and lower power when analyzing complex traits (Segura *et al.* 2012). Additionally, the model's focus on testing 1 marker at a time may have limited its ability to capture the genetic interactions of complex traits, overlooking smaller effect associations (Sandhu *et al.* 2024). Similar results were also obtained in wheat where the performance of the MLM model was found to be limited (Sandhu *et al.* 2024), also reported by Paudel *et al.* (2021) using a similar cowpea germplasm.

### Candidate genes

A total of 63 candidate genes underlying the marker-trait associations were identified using the annotated cowpea reference genome (Liang *et al.* 2024; Supplementary Table 1) and expressed in 1 or more cowpea tissues according to the LIS website (https://mines.legumeinfo.org/vignamine/begin.do). The 10 kb window upstream and downstream from the position of significant SNPs for candidate gene identification was selected based on the pattern of LD decay in our materials. Andrade *et al.* (2023), analyzing similar accessions, showed the LD decays to *r*² = 0.15 over larger distances. To narrow down the candidate gene regions, they used a 10 kb window upstream and downstream of the significant markers, to capture genes within strong LD, while reducing noise from distant loci. This window size agrees with other studies, like Xiong *et al.* (2024), who successfully used the same window to identify candidate genes for seed coat patterns in cowpea. Based on expression levels observed in seed and/or pod tissues, we have highlighted some of the most promising gene candidates that may influence the traits analyzed in this study.

In the genomic regions containing SNP 2_05101 (51,419,219 bp), associated with pod length, seed area, seed length, and seed perimeter, 2 genes (*Vigun03g319800*; *Vigun03g319900*) encode for PPR-containing proteins, already reported as an important player in seed development in several crops, including chickpea, rice, and maize (Verma *et al.* 2015; Li *et al*. 2018; Lyu *et al.* 2020). Similarly, other candidate genes related to seed shape and size are annotated as PPR proteins in common bean (Giordani *et al.* 2022).

The gene *Vigun03g320300* associated with seed weight (SNP 2_08497—51,450,779 bp) encodes to an LRR-containing protein. Interestingly, it has been described that LRR proteins play important roles in the growth and development of different tissues of plants, including the regulation of seed development in *A. thaliana* and common bean (Luo *et al.* 2005; Giordani *et al.* 2022). *Vigun02g197700*, associated with 100-seed weight (SNP 2_27488—33,282,856 bp), encodes an F-box protein (Supplementary Table 1), also known to play diverse roles in plant growth/development. It has been reported that F-box proteins control seed germination, seed size, and pod-related traits in *A. thaliana* (Majee *et al*. 2018), *Oryza sativa* (Chen *et al*. 2013), and *A. hypogaea* (Zhang *et al*. 2023), respectively.

Our analysis also identified a gene (*Vigun03g283400*) associated with seed area and seed length (SNP 2_10581–46,387,165 bp) that encodes a FRIGIDA-like protein. FRIGIDA-related genes are associated with some biological processes connected with reproduction, such as embryonic development, flowering regulation, and seed maturation (Choi *et al.* 2009; Risk *et al.* 2010; Vieira *et al*. 2019). Associated with pod length, SNP 2_20642 (1,274,892 bp) is located close to *Vigun05g016000*, a Mitogen-activated protein Kinase Phosphatase 1 (MKP1) associated with various cellular processes in *A. thaliana*, including growth, development, and stress response (Ulm *et al.* 2002; Bartels *et al.* 2009; Tamnanloo *et al.* 2018).

GWAS has been increasingly applied to investigate the genetic control of several important cowpea traits, but this is the first study to combine high-density SNPs and HTP to perform association mapping and dissect the genetic architecture of important traits. In addition to having detected a new set of SNPs, some of which are promising resources to be investigated for use in MAS, we identified several genes pinpointed as interesting candidates underlying phenotypic variation in yield component traits and seed morphology traits in cowpea. These SNP markers and genes are promising targets for genetic improvement and deserve further investigations to explore their possible roles in cowpea breeding efforts. While our study used the Cowpea iSelect Consortium Array to provide genome-wide coverage, future research could consider fine mapping approaches to refine candidate regions and develop custom markers closer to causal polymorphisms, potentially reducing genotyping costs and improving precision for breeding applications.

## Conclusions

The results from our study indicate the significance of understanding the genetic basis of key agronomic and seed morphology traits in cowpea. Through GWAS on the UCR Minicore collection, we identified 42 genomic regions that are mediating seed and yield components. The wide variation observed alongside high heritability for most traits, emphasized the potential to improve these traits in cowpea. High correlations among seed size traits suggest improvement potential through indirect selection. The integration of high-density SNP genotyping with HTP enabled the identification of promising candidate genes that can be validated and applied in MAS. Our findings offer valuable knowledge for cowpea breeding, contributing to efforts that aimed to improve productivity and consumer appeal.

## Supplementary Material

jkaf024_Supplementary_Data

## Data Availability

All phenotypic data used in this study are deposited in the Dryad Digital Repository under DOI https://doi.org/10.5061/dryad.r4xgxd2pm. The genotypic data is available in Muñoz-Amatriaín *et al*. (2021). Supplemental material available at G3 online.
