# Supplementary material for: Genome-wide association studies dissect the genetic architecture of seed and yield component traits in cowpea (Vigna unguiculata L. Walp)
Source: G3 (Bethesda). 2025 Feb 7;15(4):jkaf024. doi: 10.1093/g3journal/jkaf024 (PMC12005157; doi:10.1093/g3journal/jkaf024)
Supplement: jkaf024_Supplementary_Data [file jkaf024_supplementary_data.zip › Supplementary_Figure_Legends_G3-2025-405664.docx]

**Supplementary Figure Legends**

**Supplementary Figure S1.** Phenotypic correlation analysis among traits (upper diagonal), histograms of residuals for each trait (diagonal), and pairwise scatterplots between traits (lower diagonal), with significance levels denoted as *, **, and *** representing p < 0.05, p < 0.01, and p < 0.001, respectively.

**Supplementary Figure S2.** QQ Plots for all eight traits measured. A; Seed weight, B; Seed Area, C; Seed Length, D; Seed Width, E; Seed Perimeter, F; Yield, G; Pod Number and H; Pod Length.

**Supplementary Figure S3.** Gene ontology categories (“molecular function”, “cellular component” and “biological processes”) and sub-categories found for the annotated genes identified in the genomic regions significantly associated with pod number, 100-seed weight, pod length, seed area, seed length, seed perimeter and seed width in cowpea. Sizes of the rectangles are adjusted based on the frequency of the observed GO terms.

**Supplementary Figure S4.** Comparison of Manhattan plots generated using the MLM approach vs the FarmCPU method for four traits: Pod number, 100 Seed weight, Pod length and yield.

**Supplementary Figure S5.** Comparison of Manhattan plots generated using the MLM approach vs the FarmCPU method for four seed traits: Seed Area, Seed Length, Seed Perimeter and Seed Width.
