# Supplementary figures and images for: Genome-wide association studies dissect the genetic architecture of seed and yield component traits in cowpea (Vigna unguiculata L. Walp)
Source: G3 (Bethesda). 2025 Feb 7;15(4):jkaf024. doi: 10.1093/g3journal/jkaf024 (PMC12005157; doi:10.1093/g3journal/jkaf024)

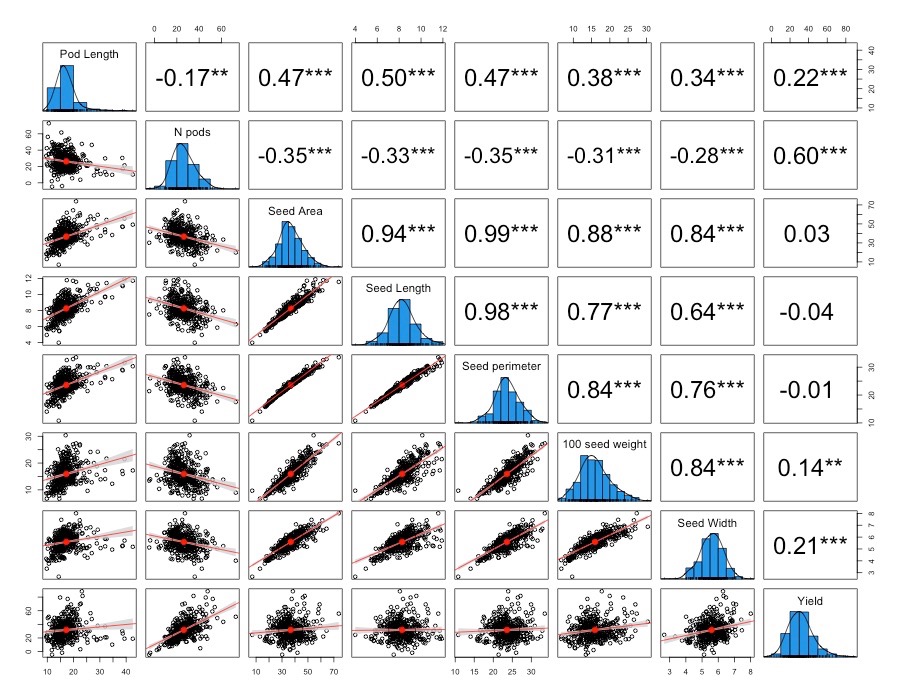

Supplement: jkaf024_Supplementary_Data [file jkaf024_supplementary_data.zip › Supplementary_Figure_S1_G3-2025-405664.jpg]

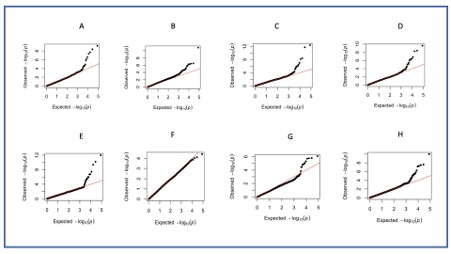

Supplement: jkaf024_Supplementary_Data [file jkaf024_supplementary_data.zip › Supplementary_Figure_S2_G3-2025-405664.jpg]

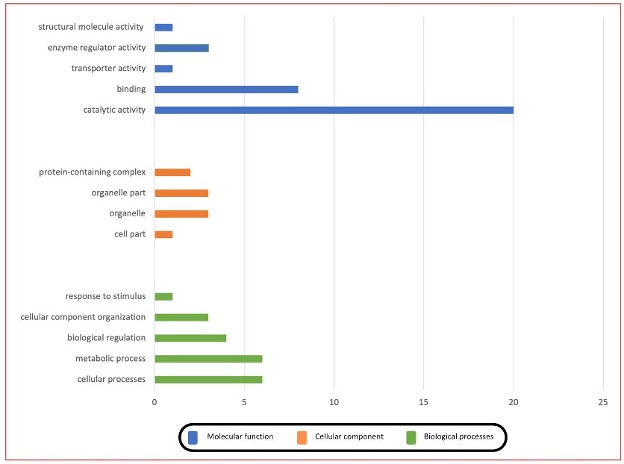

Supplement: jkaf024_Supplementary_Data [file jkaf024_supplementary_data.zip › Supplementary_Figure_S3_G3-2025-405664.jpg]

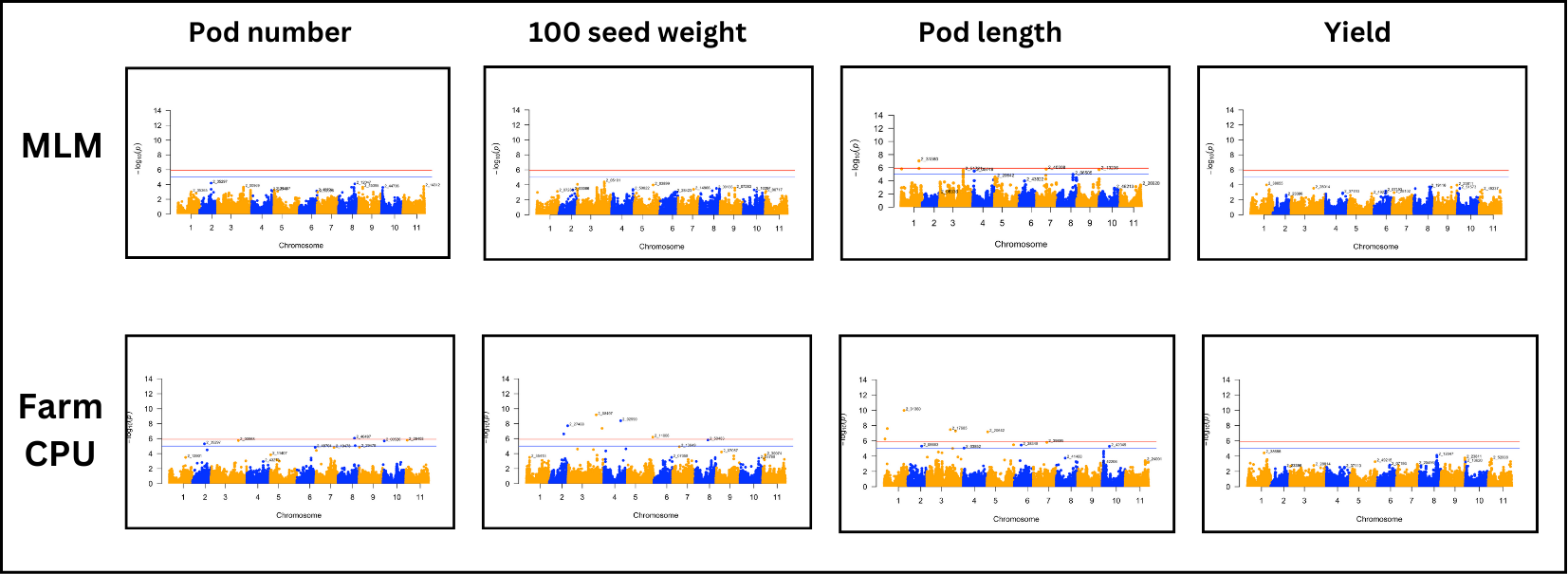

Supplement: jkaf024_Supplementary_Data [file jkaf024_supplementary_data.zip › Supplementary_Figure_S4_G3-2025-405664.png]

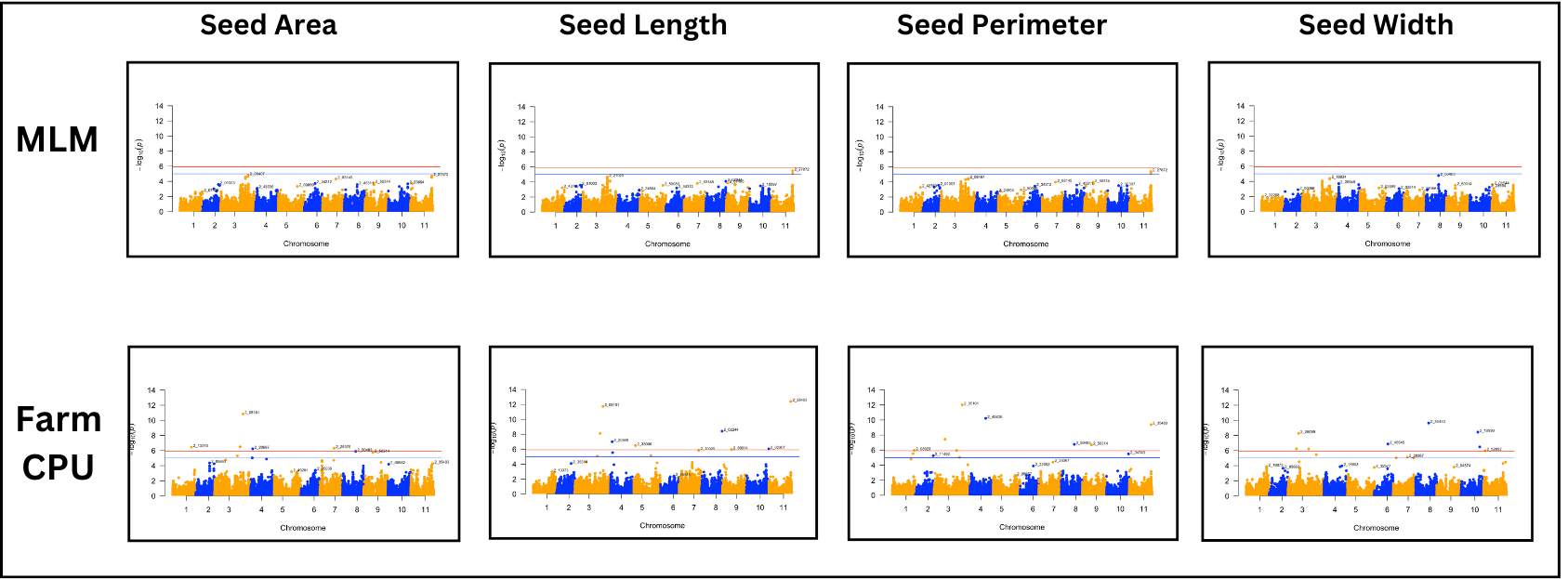

Supplement: jkaf024_Supplementary_Data [file jkaf024_supplementary_data.zip › Supplementary_Figure_S5_G3-2025-405664.png]
